# Influence of deformation banding instabilities on small scale yielding of a Mg–Nd alloy revealed by in-situ digital image correlation

**DOI:** 10.1038/s41598-023-33072-8

**Published:** 2023-04-08

**Authors:** Evgenii Vasilev, Jie Wang, Gaoming Zhu, Marko Knezevic

**Affiliations:** 1 Department of Mechanical Engineering, University of New Hampshire, Durham, NH 03824 USA; 2 National Engineering Research Center of Light Alloy Net Forming, Shanghai Jiao Tong University, Shanghai, 200240 China

**Keywords:** Engineering, Materials science

## Abstract

Propagating deformation bands are observed to accommodate the initial plasticity in an as-extruded Mg–1.5Nd alloy under tension using digital-image-correlation. The propagating bands cause an uncommon plateau in the stress–strain response of the alloy prior to restoring a common decreasing work hardening with further straining. Effects of the deformation banding and underlying plateau in the flow stress on small scale yielding are investigated during low cycle fatigue (LCF) and tension of notched specimens. Alternating formation/disappearance of deformation bands in the gauge section of as-extruded LCF specimens during testing is observed to reduce life compared to annealed specimens exhibiting no instabilities. In contrast, the bands deflect the plastic zone ahead of the notch from the principal plane orthogonal to the applied loading inducing positive effect on toughness of the alloy.

## Introduction

Alloys in which the so-called yield point phenomenon occurs exhibit a characteristic plateau stage i.e. an almost constant flow stress upon yielding^1,2^. Plastic deformation in the plateau takes place locally through instabilities such as in deformation bands, often termed as Lüders bands^3,4^. While such plastic instability phenomena are frequently observed in mild steels during tensile deformation^5,6^, they are less common in Mg alloys^7–9^. Traditionally, the inhomogeneous plastic flow in Mg alloys was largely associated with the avalanche of localized extension twining activity^10,11^. Twinning, as an important deformation mechanism in Mg, is dependent on the loading path relative to the grain crystal orientation^12–15^. Specifically, the deformation of highly textured Mg alloys in compression along the extrusion or rolling direction is dominated by extension twinning^16–18^. Twin cascades where twins in one grain stimulate twins in the neighboring grains across the grain boundary could also occur^11,19–22^. Since extension twinning causes little strain hardening in Mg alloys^23–26^, the occurrence of profuse twinning, also referred to as twin banding, could induce a plateau in the mechanical response^27^.

Instead of twinning, dislocation-induced plastic instability phenomena have recently been identified in some Mg alloys, specifically those containing rare earth elements^7,9,28–31^. Like in steels, the interaction between solute atoms and/or small precipitates and dislocations in Mg can strongly influence the flow behavior and can lead to plastic instabilities detectable at the macroscopic scale^28^. Although first described more than 150 years ago, the studies on plastic instabilities have mostly been carried out on steels^32^ and other body-centered cubic (bcc)^33^ metals. The number of studies on this subject in Mg alloys is limited. Given the growing interest in lightweight alloys^34–37^, it is now of interest to understand the nature and consequences of the plastic instability phenomena in Mg alloys.

The plastic instabilities are considered as undesirable phenomena during shaping operations because of surface irregularities they create known as stretcher-strains, Luders, or Hartman bands^38^. It remains to be determined whether any positive effects these instabilities might offer to the behavior of Mg alloys. In low-cyclic fatigue (LCF) tests, strain amplitudes are usually set below 3%^39–41^. In fracture toughness tests, the plastic zone ahead of the crack tip is used to assess the intrinsic toughness^42^. These two tests involve a small amount of plastic deformation and a localized plastic deformation, respectively. Since the plastic instability phenomena are likely to influence such small-scale yielding properties, this work here investigates LCF and toughness of the Mg–1.5Nd alloy exhibiting the observed phenomena^28^.

## Material and testing methodology

**Figure 1:**
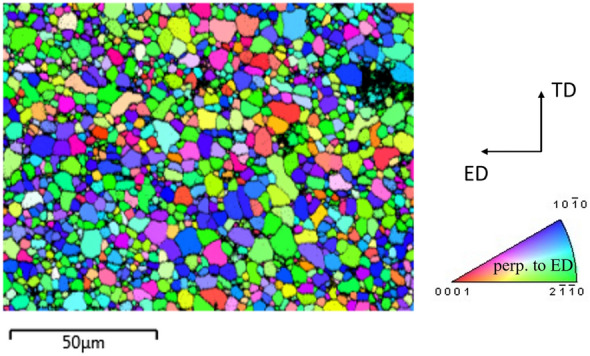
Inverse pole figure (IPF) map showing the initial microstructure of Mg-1.5Nd alloy. The sample direction perpendicular to the maps is perpendicular to ED as indicated in the standard IPF triangle. The colors in the IPF maps represent the orientation of perpendicular to ED sample axis with respect to the crystal lattice frame according to the coloring in the standard IPF triangle.

The alloy containing 1.5 wt.% Nd was cast conventionally, and then hot extruded at 300 ºC to bars of 12 mm diameter. While we used 99.95% purity of pure Mg to create the alloy, Table 1 details the composition of the alloy. Initial microstructure of the as-extruded alloy is provided in Fig. 1. The microstructure consists of equiaxed grains with an average grain size of ~ 4.3 μm predominantly oriented in a so-called ‘rare earth’ texture. More details on the microstructure derived from electron backscattered diffraction (EBSD), transmission electron microscopy (TEM), and synchrotron X-ray diffraction are provided in^28^. A few samples were subjected to short annealing at 375 °C for 15 min. The idea behind this annealing was to eliminate or at least minimize the effect of the shear band without compromise of yield strength. Since the annealing time was relatively short, it did not cause appreciable changes in the grain structure and texture compared to the as-extruded material. Therefore, a map of the annealed material is not provided. However, the annealing was sufficient to alter the solute clusters and metastable precipitates beyond the critical size to diminish the pinning of dislocations. Thus, the annealing step effectively eliminated the yield point phenomenon without compromising strength of the alloy. The dislocation pinning in the alloy is caused by small ~ 5 nm metastable precipitates and solute clusters as detailed in our earlier work^18^. The material was also annealed at 400 °C and at 485 °C for 15 min, 30 min, and 60 min and tested in tension to fracture. Results showed that the strength reduces with annealing temperature and annealing time.

**Table 1:** Content of elements in our Mg–Nd extrusion alloy, in weight percent, determined by Inductively Coupled Plasma Atomic Emission Spectroscopy (ICP-AES).

Nd	Fe	Ni	Cu	Si	Mn	Zn	Mg
1.5	0.0024	0.0082	0.0004	0.0762	0.0365%	0.0066	Bal

Flat specimens with gauge section dimensions of 5.0 mm (L) × 4.0 mm (W) × 2.0 mm (T) and round specimens with gauge dimensions of 25.0 mm (L) × 7.0 mm (D) were cut from the extrusion bars using electric discharge machining (EDM) and turning, respectively. All specimens subsequently underwent grinding surface treatment of all sides consistently using SiC papers up to 1200 grit. Additionally, a notch with radius of 1 mm was machined in a set of flat specimens. The tests were repeated at least two times to ensure the results show sufficient similarity in the mechanical characteristics and observed phenomena. The loading axis was parallel to the extrusion direction (ED). Flat samples were tested using a Gatan micro-stage, while round samples were tested using a servo-hydraulic MTS machine Landmark 270. DIC was used to record the displacement/strain/strain-rate data. To this end, a speckle pattern was applied over the surfaces using Rust-Oleum spray paint with black dots on white background. Images were captured using Point Grey camera with fixed focal length lens and extension spacers to vary the total focal length. PointGrey FlyCap2 software was used for image recoding and VIC-2D v.6 was used for postprocessing the data. The strain rate was 0.001/s for all tests in the present work.

## Results and discussion

**Figure 2:**
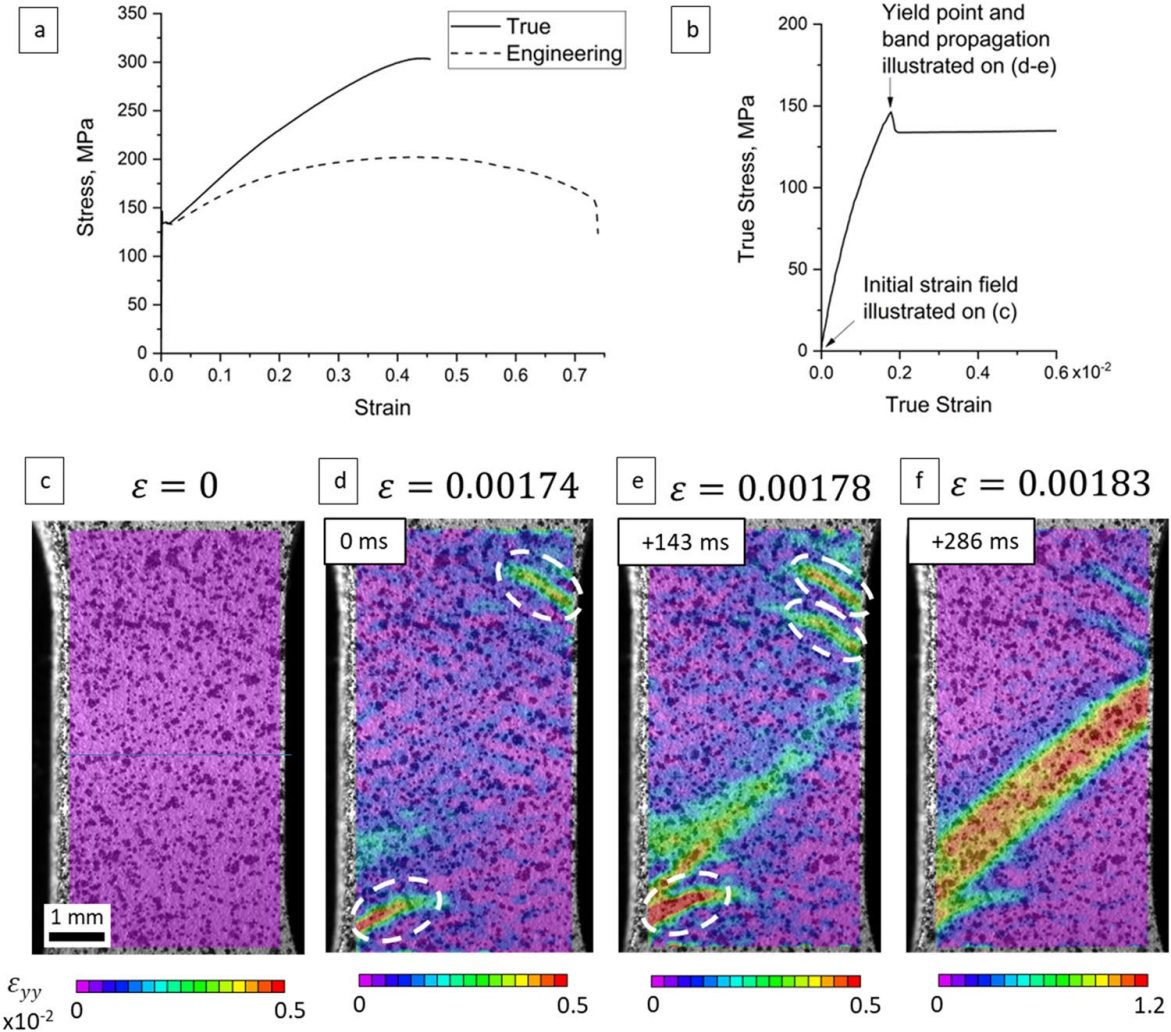
(**a**) Stress–strain curve of the Mg–1.5Nd alloy along with (**b**) a magnified portion governed by the yielding point phenomena. (**c**–**f**) DIC strain fields (ε_yy_) over the entire gauge section of the specimen in which the dashed ovals highlight tongue-shaped elasto-plastic strain localizations prior to the formation of a dominant deformation band.

We begin with showing results of simple tension tests using the flat specimens coupled with a DIC system to characterize the deformation features of plastic instability phenomena. Figure 2a shows a typical stress–strain curve of the alloy during quasi-static tensile loading. Figure 2b magnifies the portion near yielding. A plateau stage can be seen from the stress–strain curve upon yielding, which is associated with the inhomogeneous deformation of the alloy. Figure 2c–f show strain fields recorded using DIC correlating the yielding and the deformation bands developing. Tongue-shaped strain localizations develop first in the region near transition from the grip section to the gauge section of the specimen while approaching the macroscopic yielding (Fig. 2d). Upon multiple such localizations (Fig. 2e), a band propagates through the entire width of the sample at ~ 45° to the loading direction connecting the localizations from the bottom to the top (Fig. 2e). Finally, the bend predominates rising the local strain to 0.012, while the rest of the localizations fades away.

As the images in Fig. 2d–f were recorded with known time resolution of 143 ms (ms), velocity of the band propagation was estimated. As the length of the band measured using DIC was about 6 mm, the velocity can then be estimated as 6 mm in 286 ms = 0.021 m/s. This value is at least one order of magnitude lower than the velocity of twin propagation, which was earlier established to be 0.1–10 m/s^43^. While both twinning and banding are shear deformations, the difference in velocities is attributed to the difference in scales of the phenomena. Twinning is a mechanism involving shear propagation though a single grain or sub-grain, while the band involves a cascade-like ‘unpinning’ of dislocations and collective propagation of shear though a polycrystalline aggregate as shown in^28^.

**Figure 3:**
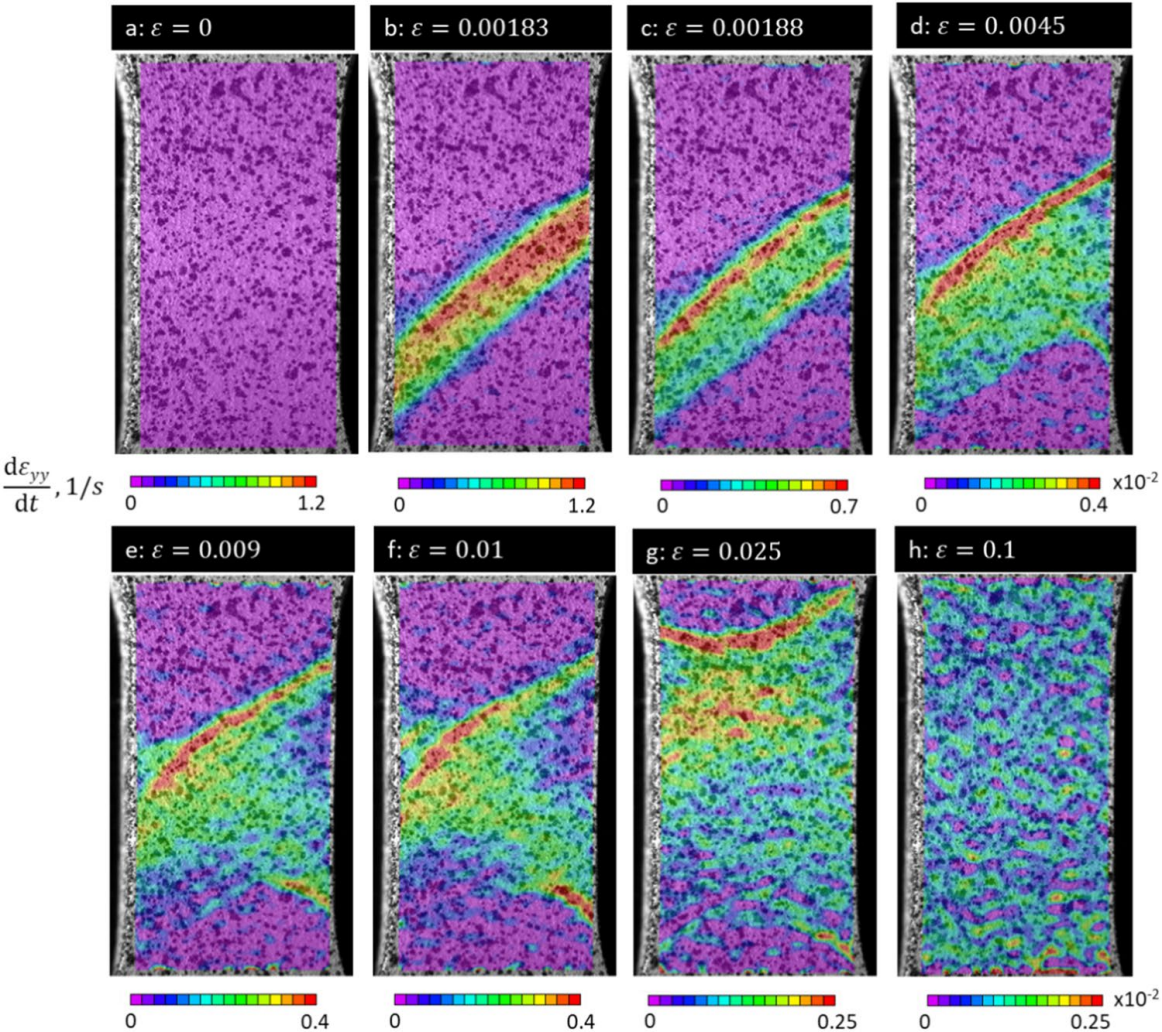
DIC strain rate fields (dε_yy_/dt) illustrating the development of plastic instabilities from (**a**) *ε* = 0 to (**b**) *ε* = 0.00183, (**c**) *ε* = 0.00188, (**d**) *ε* = 0.0045, (**e**) *ε* = 0.009, (**f**) *ε* = 0.01, (**g**) *ε* = 0.025, and (**h**) *ε* = 0.1.

To illustrate the propagation of the band under tension, we present DIC strain-rate fields (dε_yy_/dt) in Fig. 3. The figure illustrates further development of the plastic instability upon formation. Evidently, the band propagates in both directions (Fig. 3c). The instantaneous fields show that the deformation occurs between the two fronts of the band (i.e. inside the band), while the rest of the material remains elastic. Nevertheless, the strain-rate at the fronts of the band is approximately two times higher than in the interior of the band. As the band takes over the entire gauge section (Fig. 3g), the alloy restores a common decreasing strain hardening rate with further plastic straining. Therefore, deformation within the plateau is accommodated by propagation of the band. As shown in^28^, the nucleation and propagation of the band has no interference with deformation twinning. The mechanism was primarily attributed to cascade unpinning of immobilized dislocations. Such behavior of dislocations results in nucleation and propagation of the band as shown on Figs. 2f and 3b–f respectively.

**Figure 4:**
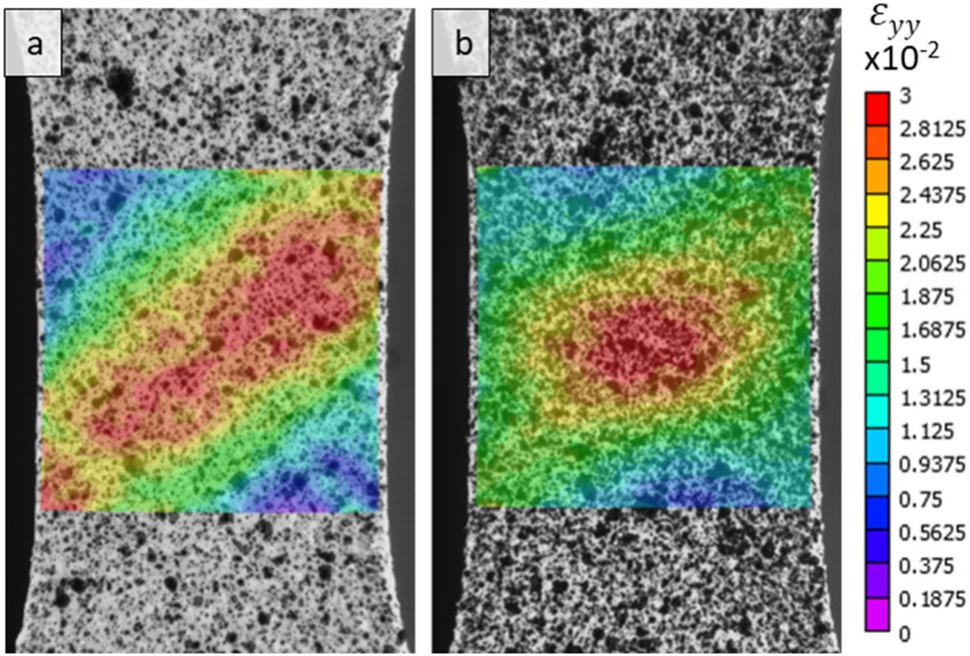
DIC strain fields ε_yy_ under tension to ~ 0.01 true strain illustrating the differences in deformation between (**a**) as-extruded Mg–1.5Nd alloy and (**b**) annealed Mg–1.5Nd alloy at 375 °C for 15 min.

Figure 4 compares strain fields developed under tension of an as-extruded sample versus a sample that underwent annealing. Annealing is known to eliminate the yield point phenomenon except in some ultrafine-grained or nano-grained materials produced by severe plastic deformation with grain sizes smaller than 1 µm^44^. The fields confirm that the non-annealed specimen deforms by a formation of a deformation band oriented ~ 45° relative to the loading direction, while the annealed specimen shows no formation of a deformation band but a regular strain localization in the middle of the gauge section due to the highest triaxiality at the center of the specimen.

**Figure 5:**
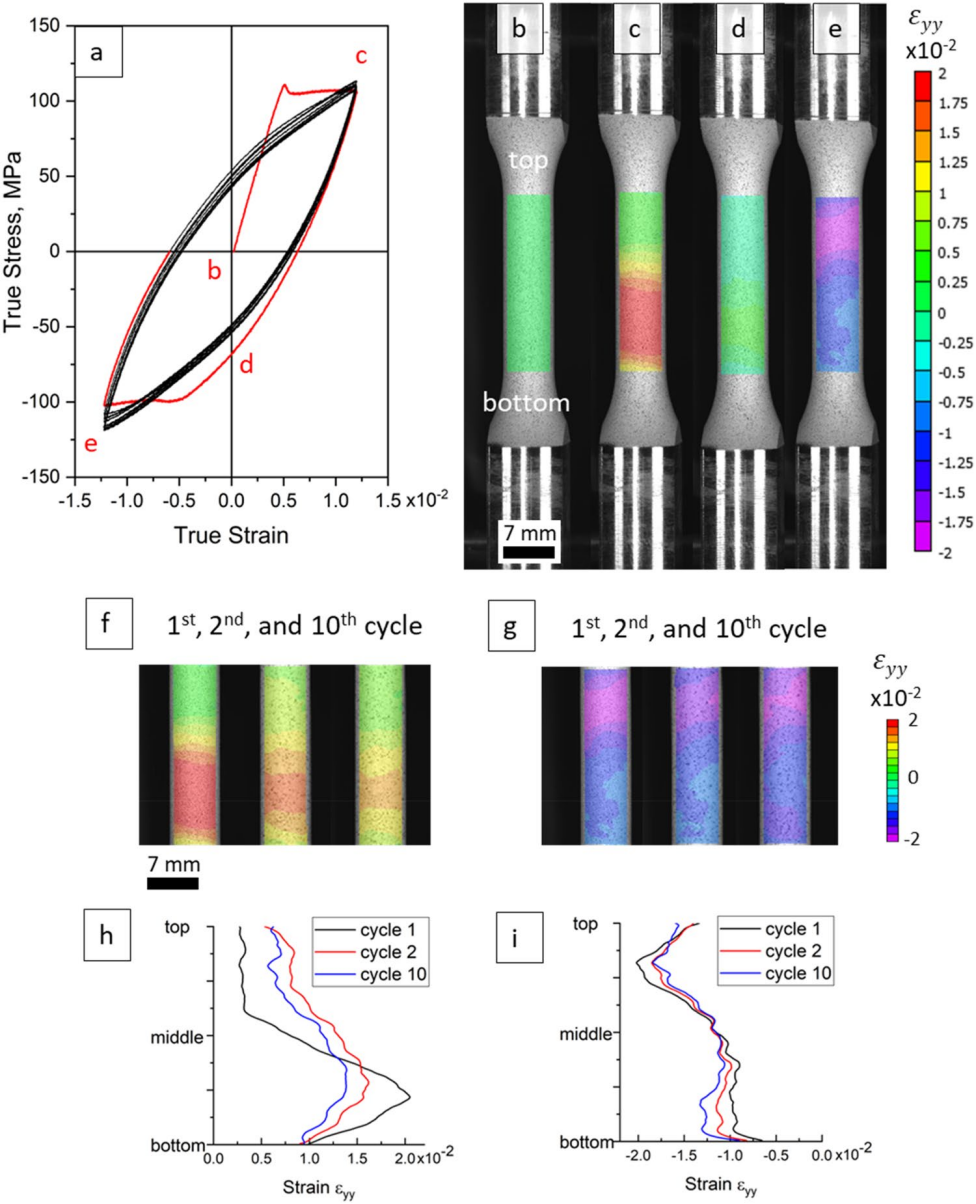
(**a**) Stress–strain curve recorded during cyclic tension–compression loading of the as-extruded alloy. DIC strain fields ε_yy_ during the 1^st^ loading cycle: initial (**b**), at maximum tension (**c**), unloaded to 0 strain (**d**), and at maximum compression (**e**). DIC strain fields ε_yy_ at maximum tension (**f**) and at maximum compression (**g**) during the 1st, 2nd, and 10th cycles. Distributions of ε_yy_ strain along the gauge section of the specimen after the 1st, 2nd, and 10th cycles at (**h**) maximum tension and (**i**) at maximum compression.

The role of deformation banding instabilities on small scale yielding of a Mg-Nd alloy is studied by carrying out LCF tests and tension of notched specimens. First, we explore the behavior of plastic instability under cyclic loading and its influence on LCF of the alloy. Figure 5 presents results of a fully reversed tension–compression LCF test for a non-annealed round specimen of Mg-1.5Nd alloy. The loading was applied with a strain amplitude of 0.0125. In Fig. 5a, the red curve corresponds to the first fully reversed cycle and the red letters b-e correspond to the DIC strain fields presented in Fig. 5b–e. The black curves in Fig. 5a correspond to subsequent cycles. The yield plateau appeared in both the first forward tension and the first reversed compression loadings, after which it disappeared. The annealed material shows no such plateau.

The deformation band appeared at the lower part of the gauge section and propagated toward the other end, reaching a local maximum of ~ 0.02 tensile ε_yy_ strain (Fig. 5c,h). The band progressively faded during the unloading process to the point where it completely vanished (Fig. 5d). Upon the compression loading, a new compressive band formed in the top part of the sample reaching a similar local maximum of − 0.02 ε_yy_ strain (Fig. 5e,i). The alternating appearance of the tensile and compressive deformation bands during the following cyclic loadings repeated in a similar pattern (Fig. 5f,g), but the extent of such plastic instability was progressively waning. After the 10th cycle, the band is ‘stabilized’ and no longer reduces in maximum strain; the repetitive appearance and disappearance of the band is observed till the fracture of the sample. This unique cyclic deformation behavior, associated with the plastic instabilities, was not reported in previous studies. Interestingly, the localized behavior of the band observed by DIC is not reflected in the stress–strain cyclic curve from the 2nd cycle onwards as there is no yield plateau in the stress–strain curve.

The observed behavior results from dislocation glide in the materials that is responsible for the presence of yielding point phenomenon. On the first loading cycle, dislocations are pinned by the metastable precipitates, and their collective unpinning causes easy yielding. Regular ‘unpinned’ glide predominates from the second cycle. After unpinning during the first cycle, the dislocations likely shear over the same path again and again during the cyclic deformation so that the precipitates offer no pinning resistance to the glide of dislocations. Essentially, dislocations glide is increasingly easier with cycles because obstacles on the same path have been overcome during previous cycle.

A series of LCF tests involving three strain amplitudes were performed on annealed and non-annealed specimens of the alloy to assess the effect of plastic instabilities on life of the alloy. Results showing the LCF life are summarized in Table 2, while a more detailed table of parameters along with plots of stress-amplitudes are shown in the supplementary material of the paper. As can be seen, the non-annealed samples deforming with the plastic instabilities have fatigue life shorter for 20–60% compared to the annealed alloy, with larger difference at lower strain amplitudes. As shown in Fig. 5, the deformation bands cause straining of about half of the gauge section to about two times higher strain level than applied instead of spreading of the strain uniformly over the entire gauge section. The latter is normal behavior of the annealed material. With each cycle, the same region of specimen becomes highly strained repeatedly while the rest of the material remains elastic. An analogy can be drawn with a known effect of mean strain on fatigue life. For a given strain amplitude, fatigue life will be the lowest for a specimen cycled in only tension (mean strain is positive), followed by cycling with mean strain equal to 0 (fully reversed tension–compression), and the highest life will be for cycling in only compression (mean strain is negative)^39,45^. Therefore, the deformation behavior with plastic instabilities negatively influences the fatigue life given that a portion of the specimen experiences only tension.

**Table 2:** LCF life for as-extruded and annealed specimens of Mg–1.5Nd alloy.

Analysis parameter	Material condition: initial as-extruded (I) versus annealed (A)	Strain amplitude, × 10^–2^		
		1.45	1.8	2.3
Number of cycles to fracture	I	779	347	140
	A	1231	475	169
Ratio of cycles to fracture between annealed samples compared to non-annealed samples		1.58	1.37	1.21

**Figure 6:**
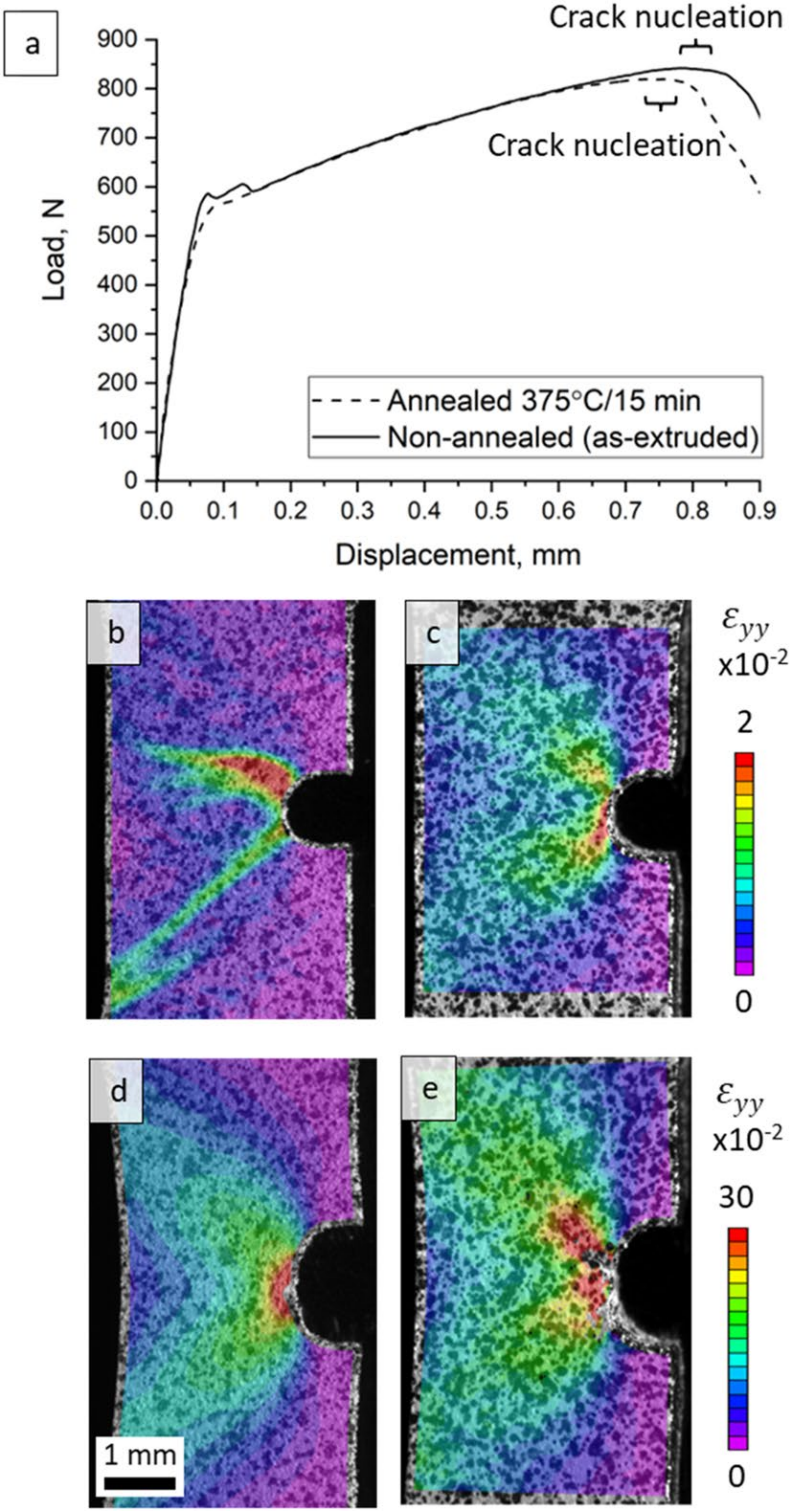
(**a**) Comparison of load–displacement (P-δ) curves recorded during tension of notched as-extruded and annealed specimens of the Mg–1.5Nd alloy. (**b**–**e**) DIC strain fields ε_yy_ illustrating the fields at the beginning of deformation (**b**, **c**) and at the beginning of crack nucleation (**d**, **e**). These are indicated by brackets in (**a**): as extruded specimens (left) and annealed specimens (right). Note the similarity of the strain fields in (d and e) with a slightly higher strain localization in the annealed specimen.

**Figure 7:**
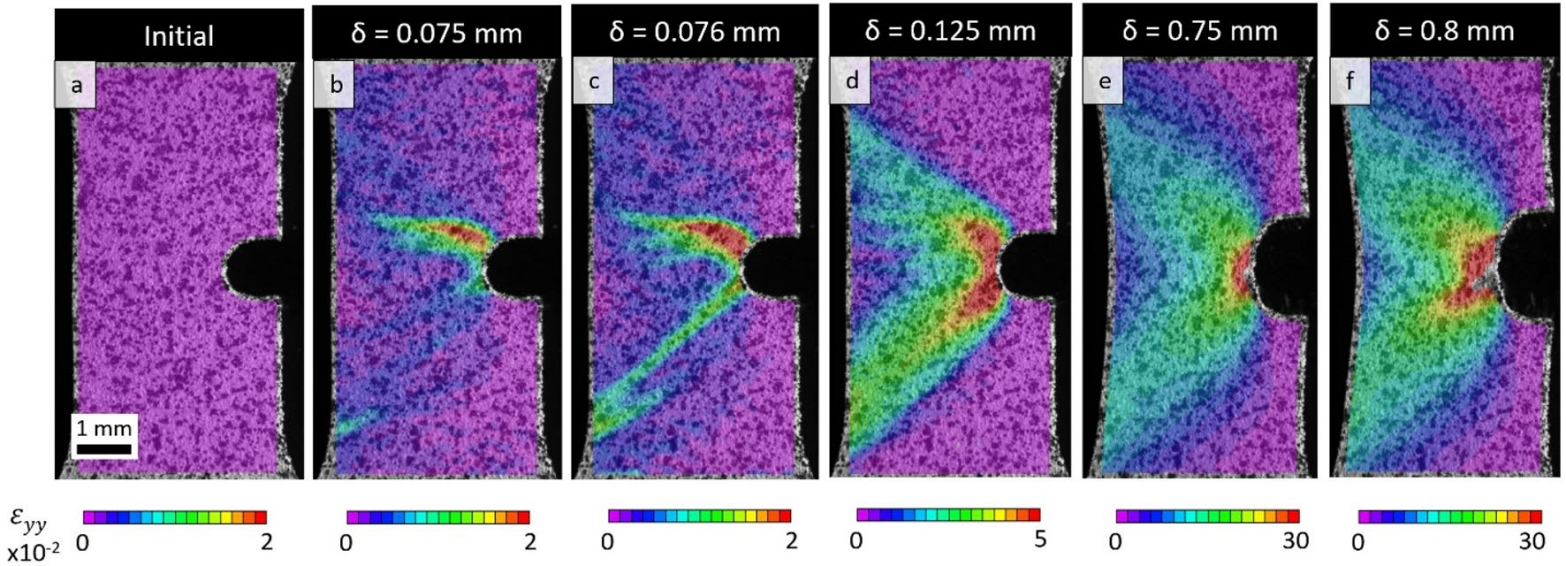
DIC strain fields (ε_yy_) during tension of a notched specimen of as-extruded Mg–1.5Nd alloy at the following displacements (δ): (**a**) δ = 0, (**b**) δ = 0.075, (**c**) δ = 0.076, (**d**) δ = 0.125, (**e**) δ = 0.75, and (**f**) δ = 0.8.

To evaluate the effect of plastic instability on toughness of the alloy, tensile tests involving notched specimens of as-extruded and annealed alloy were performed. The load–displacement curves along with a comparison between the fields for the as-extruded versus annealed specimens are provided in Fig. 6. Figure 7 presents DIC strain fields ε_yy_ for the as-extruded specimen. Similar to the fields under tension of unnotched specimens, the formation of tongue-shaped elasto-plastic localizations were observed right before the band propagation. Strain localization ahead of the notch is expected, but the irregular and asymmetric shape of it is not typical^42^. Upon reaching yielding, the first band propagates on the side of the notch opposite to the location of the localization (Fig. 7). A load drop was observed from the load–displacement curve, which is followed by a short portion of linear hardening. As a result of such instabilities, a path of least resistance is deflected from the mode I principal plane orthogonal to the applied loading direction. Afterwards, the second shear band nucleates on the other side of a notch (Fig. 7d) causing another load drop and with further increasing of strain both bands grow in width. Once both bands are fully developed, the plastic zone ahead of the notch takes a relatively symmetric shape meaning that the alloy behaves in a common way with hardening followed by emerging and propagation of a crack at the later stage of deformation.

The load–displacement curves of the notched specimens with and without annealing become overlapping after the initial yield portion confirming that the deformation banding phenomena affect primarily the early stage of the deformation near yielding. After the bands are fully propagated, the as-extruded alloy behaves like the annealed alloy without plastic instabilities. However, the crack nucleation in annealed alloy is observed earlier compared to the as-extruded alloy. Additionally, there is a slightly higher strain localization observed in the annealed specimen compared to the non-annealed one, which is more spread. Though these differences are not prominent, these can be signs of a positive effect of the bands on toughness. The property of toughness is traditionally considered to represent a combination of strength and ductility, the two properties that are mutually exclusive in general. The alloy used in the present study exhibiting an elongation of > 40% makes the yield point stage of about 2.5% insignificant in the whole plastic deformation. In contrast, if a much stronger but less ductile alloy is considered, a plastic instability could dominate the deformation and cause much larger effect on the properties, including substantial positive effect on toughness. In closing we indicate that even though the tests performed using notched specimens do not represent valid fracture toughness tests due to simplified sample geometry, the experiments performed here infer that equations of Continuum Fracture Mechanics would fail at describing the mechanical fields near the notch because of large asymmetry/heterogeneity driven by the instabilities.

## Summary and conclusions

In summary, we showed several novel experimental observations of plastic instabilities in a Mg alloy and evaluated their influence on the mechanical behavior of the alloy in a small-scale yielding regime. In particular, we observed the formation and propagation of localized deformation bands under tension of standard and notched specimens and cyclic loading. Nucleation of a deformation band under tension is preceded by a formation of tongue-shaped elasto-plastic strain localization heterogeneities. Upon nucleation, deformation bands propagate through the entire width of specimens, which is followed by the band widening with further straining. Under cyclic loading, the deformation band forms in tension but then disappears during unloading and a new band forms in a different region under compression. Such inhomogeneous deformation repeats during cyclic loading and adversely affects the fatigue life of the alloy. Nevertheless, the plastic instabilities deflect the plastic zone ahead of the notch from the principal plane orthogonal to the applied loading inducing some positive effect on the toughness of the alloy.

## Supplementary Information

Supplementary Information.

## Data Availability

The datasets used and/or analyzed during the current study available from the corresponding author on reasonable request.
