# Supplementary material for: Influence of deformation banding instabilities on small scale yielding of a Mg–Nd alloy revealed by in-situ digital image correlation
Source: Sci Rep. 2023 Apr 8;13:5767. doi: 10.1038/s41598-023-33072-8 (PMC10082783; doi:10.1038/s41598-023-33072-8)
Supplement: Supplementary file 1 — Supplementary Information. [file 41598_2023_33072_MOESM1_ESM.docx]

Supplementary material

**Influence of deformation banding instabilities on small scale yielding of a Mg-Nd alloy revealed by in-situ digital image correlation**

Evgenii Vasilev ^a^, Jie Wang ^b^, Gaoming Zhu ^b^, Marko Knezevic ^a^

^a^ Department of Mechanical Engineering, University of New Hampshire, Durham, NH 03824, USA

^b^ National Engineering Research Center of Light Alloy Net Forming, Shanghai Jiao Tong University, Shanghai 200240, China


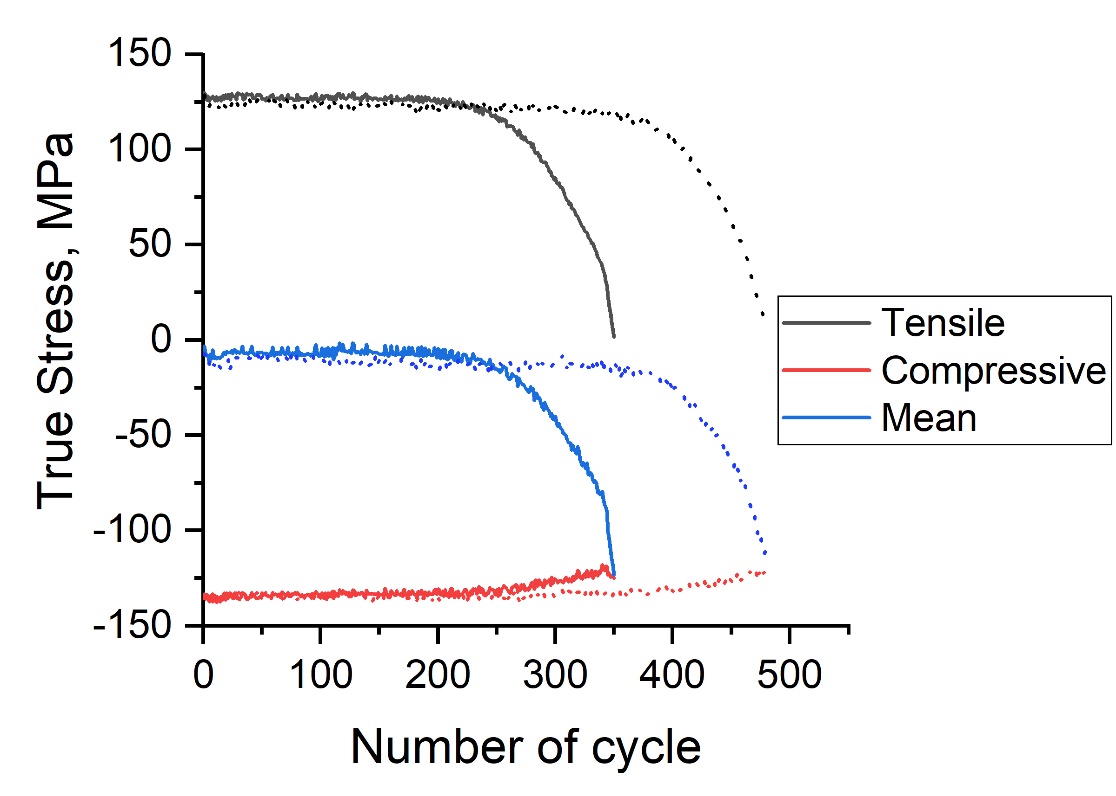


**Fig. S1**. Max tensile, max compressive, and mean true stress versus cycles during cyclic loading of as-extruded (solid lines) and annealed at 375°C for 15 minutes (dash lines) specimens of the Mg-1.5Nd alloy. The applied strain amplitude was 0.018.

Table S1– Comparison of LCF parameters for as-extruded and annealed specimens of Mg-1.5Nd alloy.

| **Analysis parameter** | **Marial condition: initial (I) or annealed (A)** | **Strain amplitude, x10^-2^** | | |
| --- | --- | --- | --- | --- |
|  |  | **1.45** | **1.8** | **2.3** |
| Number of cycles to fracture | I | 779 | 347 | 140 |
|  | A | 1231 | 475 | 169 |
| Maximum stress on the first cycle, MPa | I | -129.8 | -136.9 | -153.3 |
|  | A | -131.4 | -136.3 | -151.9 |
| Maximum stress in the mid-life, MPa | I | -129.4 | -135.7 | -157.9 |
|  | A | -120.7 | -137.4 | -157.1 |
| Maximum stress at the beginning of crack propagation, MPa | I | -124.2 | -133.8 | -158.9 |
|  | A | -121.7 | -133.9 | -159.6 |
| Mean stress on the first cycle, MPa | I | -15.2 | -7.4 | -10.2 |
|  | A | -31.5 | -9.3 | -8.4 |
| Mean stress in the mid-life, MPa | I | -6.9 | -7.4 | -19.2 |
|  | A | -15.9 | -13.9 | -20.8 |
| Mean stress at the beginning of crack propagation, MPa | I | -0.4 | -8.7 | -21.0 |
|  | A | -16.7 | -13.1 | -30.6 |
| Ratio of cycles to fracture in annealed samples compared to non-annealed samples | | 1.58 | 1.37 | 1.21 |
